# A Four-Channel Microfluidic Vascular-Wall Chip for Modeling Early Atherosclerosis-Related Endothelial Dysfunction and Evaluating Combined Anti-Inflammatory Treatment

**DOI:** 10.3390/mi17060734

**Published:** 2026-06-18

**Authors:** Xulong Wu, Yi Xu, Xiaoshuang Zhao, Xianqiang Mi

**Affiliations:** 1 School of Microelectronics, Shanghai University, Shanghai 200444, China; 20020812@shu.edu.cn; 2 Shanghai Institute of Microsystems and Information Technology, Chinese Academy of Sciences, Shanghai 200050, China; zhaoxiaoshuang@ucas.ac.cn; 3 School of Physics and Optoelectronic Engineering, Hangzhou Institute for Advanced Study, University of Chinese Academy of Sciences, Hangzhou 310024, China; 4 Human Phenome Institute, Fudan University, Shanghai 200438, China

**Keywords:** microfluidics, organ-on-a-chip, vascular-wall chip, endothelial dysfunction, early atherosclerosis, barrier permeability, macrophage recruitment, metformin, atorvastatin

## Abstract

Atherosclerosis begins with endothelial dysfunction, inflammatory activation, and immune-cell recruitment within a spatially organized vascular wall. Conventional static cultures and Transwell systems are advantageous for isolated readouts, but they fail to effectively recapitulate multicellular compartmentalization, extracellular matrix support, and dynamic perfusion within a singular platform. Here, we present a four-channel microfluidic vascular-wall chip designed to reconstitute an endothelial cell-extracellular matrix-smooth muscle cell arrangement and to model early atherosclerosis-related inflammatory endothelial dysfunction. The device comprises a perfusable endothelial channel, a collagen I hydrogel region embedded with human aortic smooth muscle cells, a cell-free matrix region, and a culture-medium supply channel. Under physiological conditions, HUVECs formed a ZO-1-positive endothelial barrier and maintained high cellular viability. Stimulation with TNF-α and IL-1β (10 ng/mL each) elevated IL-6 secretion, promoted the recruitment of THP-1-derived M0-like macrophages, disrupted ZO-1 continuity, and increased FITC-dextran permeability without causing extensive cell death. The chip was subsequently utilized to evaluate metformin and atorvastatin therapies. The combinational treatment produced a more pronounced attenuation of MCP-1 secretion than either monotherapy under the inflammatory background. While this platform does not recapitulate advanced plaque formation, lipid deposition, foam-cell formation, or disturbed arterial shear, it successfully provides a microfluidic model of early inflammatory endothelial dysfunction to facilitate mechanistic studies and preliminary anti-inflammatory drug evaluation.

## 1. Introduction

Atherosclerosis (AS) is a chronic inflammatory vascular disease characterized by endothelial dysfunction, immune-cell recruitment, smooth muscle cell remodeling, and lipid-driven plaque development [1,2]. Endothelial dysfunction represents one of the earliest pathological events and is associated with impaired barrier integrity, altered nitric oxide signaling, and aberrant endothelial activation [3,4]. Monocyte recruitment and immune-cell accumulation further amplify vascular inflammation [5,6], whereas vascular smooth muscle cell phenotypic switching contributes to lesion remodeling and plaque evolution [7]. Residual inflammatory risk remains clinically significant even when lipid-lowering therapy is administered, as demonstrated by recent anti-inflammatory cardiovascular trials [8,9]. In addition, low or oscillatory wall shear stress contributes to regional endothelial activation, permeability changes, and plaque susceptibility [10,11].

Animal models and conventional in vitro systems have contributed substantially to AS research, yet both possess distinct limitations. Static two-dimensional endothelial or smooth muscle cell cultures are simple and reproducible, but fail to provide three-dimensional extracellular matrix (ECM) support, dynamic flow, and spatially organized cell–cell communication. Organ-on-a-chip systems offer a promising alternative by integrating microfluidic perfusion, controlled tissue interfaces, and real-time imaging within microscale devices [12,13]. For vascular disease modeling, recent organ-on-chip studies and reviews emphasize the importance of geometry, mechanical stimulation, tissue interfaces, and relevant human cell sources [14,15].

Several AS-related microfluidic models illustrate the progress of this field. Reviews of in vitro AS models and AS-on-chip systems have highlighted the need to incorporate vascular-wall architecture, ECM support, barrier assays, immune-cell recruitment, and disease-specific readouts [16]. Early AS and arterial wall-on-chip platforms have been employed to examine endothelial inflammation, smooth muscle cell migration, leukocyte-endothelial interactions, and permeability changes [17]. More recent plaque- or arterial-event-oriented platforms further demonstrate that 3D organization, lipid/foam-cell components, and hemodynamic cues are critical for modeling AS beyond a general inflammatory state [18,19]. Recent microfluidic and cardiovascular organ-on-chip reviews also stress that authors should clearly state which pathological modules are included and which remain outside the model scope [20,21].

In response to these considerations, the present study develops a four-channel microfluidic vascular-wall chip specifically tailored to model early AS-related inflammatory endothelial dysfunction rather than a complete plaque-on-chip model. The chip reconstitutes a layered endothelial cell-ECM-smooth muscle cell arrangement and supports macrophage recruitment, cytokine readouts, ZO-1 imaging, and FITC-dextran permeability quantification. The platform was subsequently employed to evaluate the anti-inflammatory effects of metformin, atorvastatin, and their combined treatment. The main design features and limitations of this model compared with representative in vitro vascular inflammation and AS-related models are summarized in Table 1 [22,23]. It is worth noting that this platform specifically isolates the pre-lesional, cytokine-driven endothelial priming phase of early atherogenesis, establishing the pathological baseline before lipid deposition and foam cell development occur.

## 2. Materials and Methods

### 2.1. Microfluidic Chip Design and Fabrication

The four-channel microfluidic device was fabricated using standard soft lithography and PDMS replica molding [24,25,26]. Briefly, SU-8 photoresist was patterned on a silicon wafer to generate a master mold. Polydimethylsiloxane (PDMS; Sylgard 184, Dow Corning, Midland, MI, USA) and curing agent were mixed at a mass ratio of 10:1, degassed, poured onto the mold, and cured at 80 °C for 2 h. The cured PDMS slab was peeled off, inlet and outlet ports were punched, and the device was bonded to a glass slide after oxygen plasma treatment. The chip contained four parallel functional regions: an endothelial cell channel (800 µm × 150 µm), a smooth muscle cell channel (800 µm × 150 µm), a central collagen I hydrogel channel (100 µm × 150 µm), and a medium supply channel (800 µm × 150 µm). The straight co-culture region was approximately 10 mm long. Before cell culture, devices were sterilized, rinsed with PBS, and equilibrated with complete medium.

### 2.2. Cell Culture and Macrophage Differentiation

Human umbilical vein endothelial cells (HUVECs), human aortic smooth muscle cells (HASMCs), and THP-1 human monocytic leukemia cells were utilized. HUVECs were maintained in endothelial cell medium, HASMCs were cultured in high-glucose DMEM, and THP-1 cells were expanded in RPMI-1640 medium. Complete media were supplemented with 10% fetal bovine serum (FBS; Gibco, Thermo Fisher Scientific, Waltham, MA, USA) and 1% penicillin-streptomycin (P/S; Gibco, REF 15140-122). All cells were cultured at 37 °C in a humidified incubator with 5% CO_2_. THP-1 cells were differentiated into adherent M0-like macrophages by treatment with 100 ng/mL phorbol 12-myristate 13-acetate (PMA; Sigma-Aldrich, Burlington, MA, USA) for 24 h, followed by PBS washing and recovery in fresh complete medium for 24 h prior to experimental use. PMA-based THP-1 differentiation has been widely used for macrophage-like cell generation, although protocol-dependent variation should be considered [27,28]. Recent optimization and cell-line comparison studies further support the need to report PMA dose, treatment duration, and recovery conditions [29,30].

### 2.3. Construction of the Four-Channel Vascular-Wall Chip

The model was assembled using a sequential matrix assembly, barrier seeding, and immune cell introduction protocol. To reconstruct the medial compartment, HASMCs were suspended at 1 × 10^6^ cells/mL in 6 mg/mL rat tail collagen I (Corning or equivalent) and introduced into the SMC-associated hydrogel channel. The collagen was allowed to polymerize at 37 °C for 30 min. The adjacent endothelial channel was coated with 200 µg/mL collagen I for 1 h to enhance cell attachment. HUVECs were then introduced at 1 × 10^5^ cells/mL, and the chip was tilted at an angle greater than 45° for 1 h to promote uniform cell sedimentation along the lateral collagen interface. After seeding, the devices were maintained under static attachment conditions prior to being connected to perfusion culture.

### 2.4. Perfusion Culture and Shear Stress Estimation

The endothelial channel was continuously perfused during inflammatory induction and drug intervention utilizing the low-flow perfusion setup established in related organ-on-chip experiments. The working flow rate was set to approximately 2 µL/min. For a rectangular microfluidic channel, under the classical thin-channel fluid mechanics assumption where the channel height is significantly smaller than its width (h << w), the boundary effects of the vertical side-walls on the velocity profile are minimized. Consequently, the maximum wall shear stress (τ) acting along the centerline of the monolayer can be closely approximated using Equation (1):

τ = 6 μQ/(wh^2^), (1)

where τ denotes the wall shear stress, μ is the fluid viscosity, Q represents the volumetric flow rate, w is the channel width, and h is the channel height. Using μ ≈ 0.001 Pa·s, Q = 2 µL/min, w = 800 µm, and h = 150 µm, the calculated shear stress was approximately 0.011 Pa (0.11 dyn/cm^2^). Although the applied shear stress is lower than typical physiological arterial levels, this continuous perfusion profile was carefully selected as a deliberate experimental design balancing technical feasibility and pathophysiological relevance. Structurally, in this four-channel configuration, HUVECs are seeded against a vertical 3D collagen I hydrogel interface. Lower initial flow rates minimize excessive hydrodynamic drag forces, thereby preventing potential cellular delamination or matrix border collapse and ensuring high cell viability. Consequently, the present flow condition is considered suitable for modeling inflammation-associated endothelial dysfunction and evaluating preliminary anti-inflammatory interventions within the scope of this study. The culture medium was refreshed or recirculated every 24 h depending on the specific experiment, and effluents were collected for subsequent cytokine assays [10,11].

### 2.5. Inflammatory Induction and Drug Intervention

After 24 h of culture, upon confirming the formation of a confluent monolayer via bright-field observation and ZO-1 staining, inflammatory induction and drug treatment were initiated. Recombinant human TNF-alpha and IL-1beta were administered at 10 ng/mL each. This concentration was selected to induce endothelial inflammatory activation while avoiding confounding interpretations from severe cytotoxic injury; similar ng/mL-level stimulation has been previously reported to induce endothelial adhesion molecule expression, monocyte adhesion, cytokine release, and barrier disruption in endothelial models. Because the current dataset does not include a formal dose–response experiment, the selected concentration should be interpreted as the working inflammatory activation condition rather than an optimized pathological dose. Four inflammatory conditions were established: physiological control, TNF-alpha stimulation, IL-1beta stimulation, and TNF-alpha + IL-1beta co-stimulation. Each inflammatory background was further divided into four drug-treatment subgroups: vehicle control, metformin monotherapy (5 mM), atorvastatin monotherapy (1 µM), and metformin + atorvastatin combined treatment. Treatments were applied under perfusion for 24 h. Effluents were collected and stored at −80 °C for ELISA analysis, while the chips were retained for imaging and barrier-function assays.

### 2.6. Dynamic Macrophage Recruitment Assay

To assess immune-cell recruitment, differentiated M0-like macrophages were introduced into the endothelial channel following inflammatory induction. The cells were allowed to interact with the endothelial barrier under perfusion, and recruited or adherent macrophages were imaged in predefined fields of view. For each chip, three fields of view were analyzed and averaged prior to statistical analysis. Three independent chips were used for each group unless otherwise indicated. To ensure rigorous biological replication, these independent chips were fabricated, hydrogel-assembled, and cell-seeded across completely separate experimental batches, rather than utilizing multiple regional fields of view within a single device as technical duplicates.

### 2.7. Cell Viability and Immunofluorescence Staining

Cell viability was evaluated using a LIVE/DEAD assay. Prior to staining, the microchannels were rinsed with prewarmed PBS for 10 min. Subsequently, a Calcein-AM and ethidium homodimer-1 working solution was introduced into the channels and incubated at 37 °C in the dark for 30 min. For ZO-1 immunofluorescence staining, cells were fixed with 4% paraformaldehyde for 20 min, permeabilized with 0.1% Triton X-100 for 10 min, and blocked with 5% BSA for 1 h. The samples were then incubated with an anti-ZO-1 primary antibody overnight at 4 °C, followed by incubation with a fluorescent secondary antibody and DAPI. Fluorescence images were captured using an inverted fluorescence microscope. Since confocal Z-stack images were not acquired in the present study, the resulting ZO-1 data are described conservatively as evidence of junctional organization rather than definitive proof of a three-dimensional endothelial barrier architecture.

### 2.8. FITC-Dextran Barrier Permeability Assay

FITC-dextran (70 kDa; 100 µg/mL in serum-free medium) was employed to assess endothelial barrier permeability. Tracer-based permeability assays are commonly utilized in endothelial barrier and organ-on-chip studies, including inflammatory barrier models [31]. The tracer solution was perfused into the endothelial channel, and fluorescence images were acquired at the initial time point and at 3 min intervals for up to 30 min. The apparent permeability coefficient Papp (cm/s) was calculated from fluorescence intensity changes using Equation (2):

(2) $$P=(\frac{1}{\left(ICHt1-ITt1\right)})\times \frac{\left(ITt2-ITt1\right)}{\left(\Delta t\right)}\times \frac{VT}{A}cm/s$$

where ICHt1 is the fluorescence intensity in the endothelial channel at the initial time point; ITt1 and ITt2 are the fluorescence intensities in the target hydrogel/central region at the initial and final time points, respectively; Δt is the time interval; VT is the volume of the target region; and A is the effective endothelial barrier area.

### 2.9. ELISA Quantification

IL-6 and MCP-1 concentrations in the collected effluents were quantified using human ELISA kits following the manufacturer’s instructions. Samples were thawed on ice, centrifuged to remove suspended cells or debris, and added to antibody-coated plates. Absorbance at 450 nm was measured with a microplate reader (Thermo Fisher Multiskan or equivalent), and absolute concentrations were determined from standard curves.

### 2.10. Statistical Analysis

All quantitative data were obtained from at least three independent chip experiments unless otherwise indicated. Fluorescence images were analyzed using Fiji/ImageJ (version 1.53t). Cell viability was calculated as the ratio of Calcein-AM-positive cells to total cells. Macrophages were manually counted within the endothelial channel. For macrophage recruitment, three fields of view from each chip were first averaged to generate one biological replicate value per chip. ZO-1 continuity was evaluated via fluorescence intensity distribution and positive-area analysis. ELISA data were calculated from standard curves. Data are presented as mean ± SD. One-way ANOVA followed by Dunnett’s or Tukey’s post hoc test was applied for multiple comparisons, and unpaired two-tailed *t*-tests were utilized for two-group comparisons where appropriate. *p* < 0.05 was considered statistically significant.

## 3. Results

### 3.1. Design and Characterization of the Four-Channel Vascular-Wall Chip

**Figure 1:**
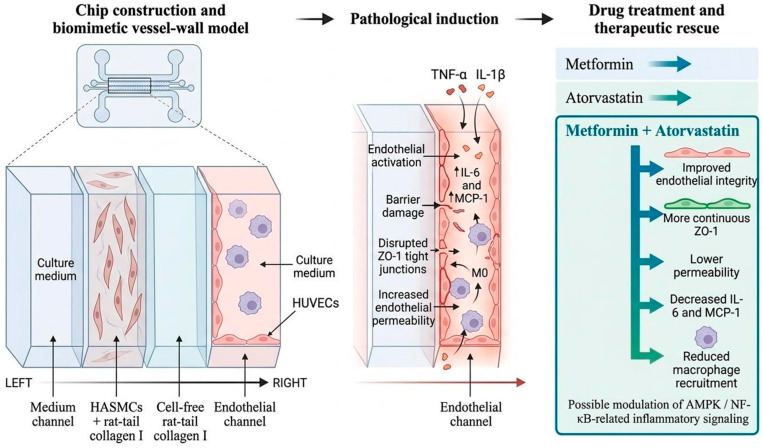
Study design and working concept of the four-channel vascular-wall chip. The platform reconstructs an endothelial cell-extracellular matrix-smooth muscle cell arrangement, induces early AS-related inflammatory endothelial dysfunction with TNF-alpha/IL-1beta, and evaluates metformin, atorvastatin, and combined treatment. The schematic diagram was technically rendered and finalized using BioRender.com.

**Figure 2:**
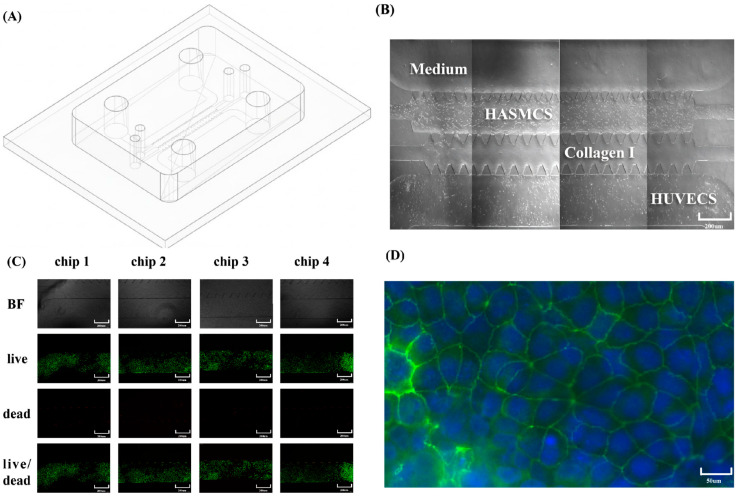
Construction and physiological validation of the four-channel vascular-wall chip. (**A**) Schematic illustration of the chip geometry. (**B**) Bright-field images showing cellular and hydrogel compartments. (**C**) Live/Dead staining in representative chips. (**D**) ZO-1 immunofluorescence showing endothelial junction formation. Scale bars are indicated in the panels.

The microfluidic device was designed to spatially separate the culture-medium channel, SMC-laden collagen I hydrogel, cell-free collagen I matrix, and endothelial channel (Figure 1). This arrangement enabled an EC-ECM-SMC architecture while preserving access for perfusion and imaging. Bright-field images demonstrated cell attachment and spreading within the corresponding channels, and Live/Dead staining indicated high cell viability under physiological culture conditions. ZO-1 staining was continuous along endothelial cell–cell borders, confirming the formation of an endothelial barrier suitable for permeability testing (Figure 2).

### 3.2. Inflammatory Stimulation Promotes Macrophage Recruitment and Cytokine Secretion

**Figure 3:**
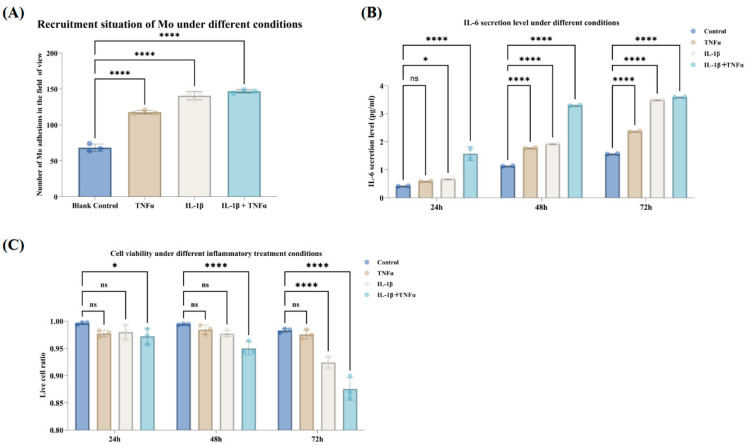
Inflammation-induced macrophage recruitment, IL-6 secretion, and viability changes. (**A**) Quantification of recruited M0-like macrophages under control, TNF-alpha, IL-1beta, and combined stimulation. For each chip, three fields of view were quantified and averaged before statistical analysis; data are presented as mean ± SD from three independent chips. (**B**) IL-6 secretion in chip effluents over time. (**C**) Live-cell ratio under different inflammatory conditions. Statistical significance was determined by one-way ANOVA with Dunnett’s test for panel (**A**) and by grouped comparisons for panels (**B**,**C**) where applicable. ns, not significant; * *p* < 0.05; **** *p* < 0.0001.

Inflammatory stimulation increased the recruitment of M0-like macrophages to the endothelial side compared with the physiological control, with the most pronounced recruitment observed under combined TNF-alpha + IL-1beta stimulation (Figure 3A). IL-6 secretion also increased over time, indicating inflammatory activation of the chip microenvironment (Figure 3B). Live/Dead staining showed that cell viability remained relatively high across groups, supporting the interpretation that the observed inflammatory and barrier changes were not simply caused by extensive cytotoxicity (Figure 3C).

### 3.3. Inflammatory Stimulation Impairs Endothelial Barrier Integrity

**Figure 4:**
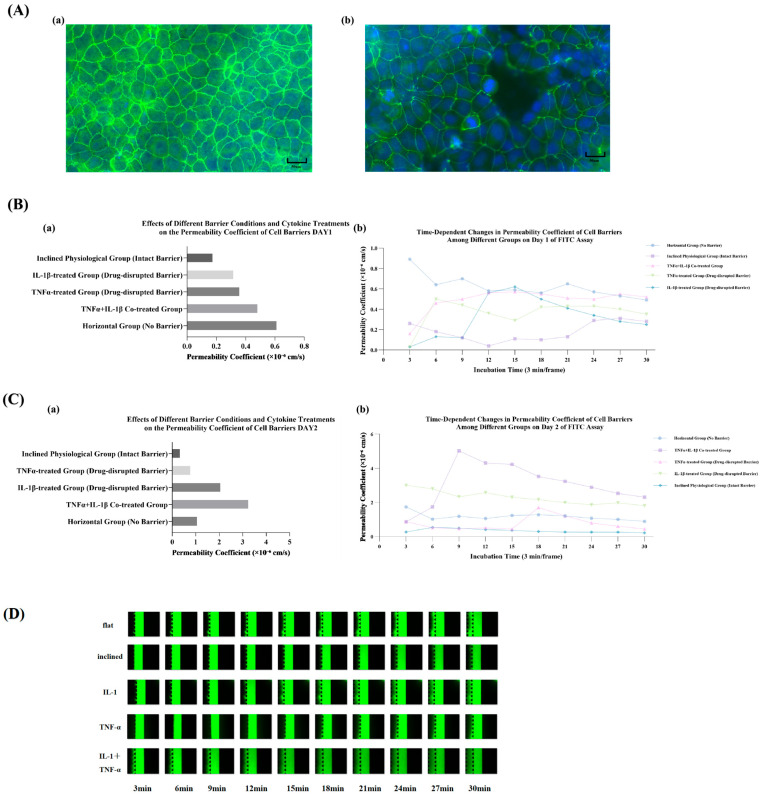
Inflammatory stimulation impairs endothelial barrier integrity. (**A**) Representative ZO-1 immunofluorescence staining showing (**a**) continuous junctional organization under physiological conditions, and (**b**) discontinuous and fragmented endothelial tight junctions under inflammatory stimulation. (**B**) FITC-dextran barrier permeability evaluation on Day 1: (**a**) apparent permeability coefficients across different barrier conditions and cytokine treatments, and (**b**) time-dependent changes in the permeability coefficient of cell barriers among different experimental groups over a 30 min incubation period. (**C**) FITC-dextran barrier permeability evaluation on Day 2: (**a**) apparent permeability coefficients across different barrier conditions and cytokine treatments, and (**b**) time-dependent changes in the permeability coefficient of cell barriers among different experimental groups over a 30 min incubation period. (**D**) Time-lapse FITC-dextran fluorescence images. Scale bars and statistical annotations are indicated in the panels.

ZO-1 immunofluorescence revealed a continuous junctional pattern in the physiological group, whereas inflammatory stimulation caused discontinuous and fragmented ZO-1 staining (Figure 4A). FITC-dextran permeability measurements further demonstrated increased macromolecular leakage in inflammatory groups compared with the intact-barrier control (Figure 4B,C). Time-lapse FITC-dextran images supported these quantitative trends (Figure 4D). Together, the structural and functional data indicate that the chip effectively models early inflammatory endothelial barrier dysfunction.

### 3.4. Combined Metformin and Atorvastatin Treatment Reduces MCP-1 Secretion

**Figure 5:**
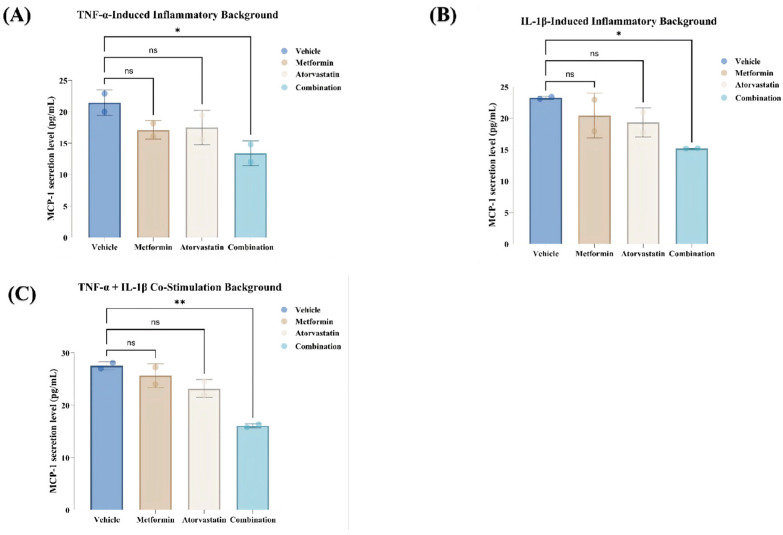
Comparative evaluation of therapeutic rescue on MCP-1 chemokine secretion under controlled inflammatory backgrounds. The quantitative ELISA data were regrouped into three standalone bar charts to directly assess drug efficacy against distinct pathological baselines: (**A**) TNF-alpha single stimulation, (**B**) IL-1 beta single stimulation, and (**C**) TNF-alpha + IL-1beta co-stimulation. Within each controlled inflammatory background, the anti-inflammatory capacities of Metformin monotherapy (5 mM), Atorvastatin monotherapy (1 µM), and Metformin + Atorvastatin combined treatment are compared against the corresponding untreated inflammatory Vehicle control. Data represent the mean ± SD from three independent chip replicates (*n* = 3). Statistical evaluations were performed via one-way ANOVA followed by Tukey’s post hoc multiple comparison tests to isolate specific inter-group drug differences (ns = not significant, * *p* < 0.05, ** *p* < 0.01).

MCP-1 was selected as a chemokine readout because it is closely related to monocyte/macrophage recruitment during vascular inflammation [5]. In the absence of drug treatment, inflammatory stimulation (TNF-alpha, IL-1beta, or their combination) markedly elevated MCP-1 secretion compared to the baseline physiological control (Normal group). To systematically evaluate the therapeutic efficacy under identical pathological conditions, the data were analyzed within each distinct inflammatory background using one-way ANOVA followed by Tukey’s post hoc test (Figure 5A–C). Under both the TNF-alpha (Figure 5A) and IL-1beta (Figure 5B) single-stimulation baselines, metformin or atorvastatin monotherapy decreased MCP-1 concentrations to varying degrees.

Most notably, under the severe combined TNF-alpha + IL-1beta co-stimulation environment (Figure 5C), where MCP-1 reached its highest baseline level (~27.55 pg/mL), single drug treatments showed limited or variable protective effects, whereas the combined metformin and atorvastatin treatment demonstrated a significant, robust reduction in MCP-1 secretion compared to both the vehicle control and monotherapy groups (*p* < 0.05). This pronounced containment under dual-cytokine challenge highlights the superior anti-inflammatory advantage of the combination regimen over individual therapies in a highly inflamed vascular microenvironment. Because formal drug-interaction modeling was not performed, the result is described as a combined inhibitory effect rather than strict pharmacological synergy.

## 4. Discussion

This study presents a four-channel microfluidic vascular-wall chip that reconstitutes an EC-ECM-SMC arrangement and supports the modeling of early AS-related inflammatory endothelial dysfunction. Compared with static culture, this platform provides spatial compartmentalization, a collagen I matrix support layer, a perfusable endothelial channel, immune-cell recruitment assessment, cytokine quantification, and FITC-dextran barrier-function readouts. These features partially fulfill the need for controllable in vitro vascular-wall models while keeping the platform simple enough for preliminary drug-response assessment. Importantly, the adopted width of the central cell-free collagen I matrix channel (100 µm) bears prominent anatomical and transport relevance. In native human macrovessels, the thickness of the internal elastic lamina and intimal layer—the foundational mass-transport boundary separating the endothelial monolayer from the smooth muscle compartment—typically spans a tens-of-micrometer scale during early vascular remodeling stages. Retaining a 100 µm gel width preserves realistic paracrine diffusion gradients for secreted cytokines and replicates the physical matrix migration scales encountered during transendothelial macrophage recruitment, aligning closely with validated biomimetic arterial-wall platforms [17,32].

The pathological framing of this disease model is intentionally conservative. Atherosclerosis is not merely a general inflammatory state; it involves lipid retention, oxidized LDL uptake, foam-cell formation, inflammatory and fibrotic remodeling, calcification, plaque growth, and hemodynamic contributions [1,2]. The present chip captures an early inflammatory endothelial dysfunction module rather than a complete plaque-on-chip phenotype. This distinction is particularly relevant because HUVECs are venous endothelial cells and do not fully reproduce coronary or aortic endothelial biology. Nevertheless, the combination of HUVECs, HASMCs, collagen I, and THP-1-derived macrophages provides a useful first-generation model for studying barrier disruption, cytokine release, and immune-cell recruitment under controlled microfluidic conditions [18,19].

TNF-alpha and IL-1beta were administered at 10 ng/mL each throughout the study. This concentration falls within a commonly used ng/mL-level range for endothelial inflammatory activation and is more suitable for cytokine-induced activation than µg/mL-level injury conditions that may cause nonspecific barrier disruption [32,33]. Because the current dataset does not include a formal dose–response experiment, the conclusion is limited to inflammatory activation under the selected stimulation condition. Future work should compare low- and high-dose cytokine conditions to distinguish endothelial activation from cytotoxic barrier disruption.

The perfusion parameters were delineated to define the vascular microenvironment. Based on the channel geometry and a working flow rate of approximately 2 µL/min, the estimated endothelial wall shear stress was approximately 0.11 dyn/cm^2^. This condition provides continuous low-flow exchange and supports dynamic exposure, but it should not be characterized as physiological arterial shear. In evaluating the current platform’s limitations, exposure to sub-physiological shear inevitably influences shear-dependent processes; under sub-physiological flow, endothelial cells retain a cobblestone morphology and exhibit reduced mechanosensitive alignment compared to the elongated morphology and reinforced tight-junction assembly found under physiological flow. However, continuous perfusion provides a dynamic microenvironment that more closely resembles in vivo vascular conditions than conventional static culture systems. Previous clinical and hemodynamic studies have demonstrated that chronically low or oscillatory shear environments (<1–2 dyn/cm^2^) are closely associated with endothelial activation, increased baseline permeability, and enhanced inflammatory responsiveness, which are localized key features of early atherosclerosis-prone vascular regions (such as bifurcations and inner curvatures). While this mechanical baseline yields a more susceptible barrier function, it successfully establishes a highly responsive analytical window that is exceptionally suited for capturing pronounced cytokine-induced barrier damage and determining the relative therapeutic rescue effects during preliminary anti-inflammatory drug evaluation. Future versions of the platform should introduce controlled pulsatile or disturbed shear profiles, use arterial endothelial cells such as HAECs or HCAECs, and incorporate oxLDL/lipid deposition and foam-cell readouts to strengthen AS-specific relevance [10,11].

The drug-evaluation data suggest that combined metformin and atorvastatin treatment reduces MCP-1 secretion more strongly than either monotherapy. This observation is consistent with reported anti-inflammatory actions of metformin through AMPK/NF-kappaB-related pathways [34,35]. Although the working concentration of metformin (5 mM) applied in this acute study exceeds typical long-term clinical serum levels, such millimolar ranges are standard and widely validated in short-term in vitro assays to reliably trigger downstream signaling responses within brief experimental windows [34]. Metformin has also been linked to vascular smooth muscle cell modulation and macrophage regulation, which are both relevant to vascular-wall inflammation [35,36]. The finding is also consistent with the vascular protective and anti-inflammatory effects broadly reported for statins [37,38]. Atorvastatin has further been reported to suppress endothelial inflammatory responses and monocyte migration-related pathways [39]. However, downstream pathway measurements and formal synergy analysis were not performed. Therefore, the conclusion is limited to a combined inhibitory effect on MCP-1 secretion. Additional readouts, including ZO-1 rescue, permeability rescue, ICAM-1/VCAM-1 expression, NF-kappaB pathway analysis, and macrophage phenotype markers, would strengthen the pharmacological interpretation.

This study presents several limitations. First, confocal Z-stack imaging was not available; consequently, the current ZO-1 data support junctional organization but cannot fully prove the three-dimensional endothelial barrier architecture. Second, the model does not include arterial endothelial cells, oxLDL stimulation, foam-cell formation, calcification, or long-term plaque remodeling. Third, the low-flow condition does not replicate physiological arterial shear or disturbed flow. Fourth, the device does not yet integrate real-time TEER or electronic barrier sensing, although such modules could further enhance future permeability assessment [40]. These limitations are explicitly stated to position the platform as an early vascular inflammation and endothelial barrier dysfunction model.

Recent microfluidic AS studies have begun to incorporate foam-cell formation, intimal-lumen architecture, and stem-cell-derived vascular modules, which clarify the next experimental targets for improving the present model [41,42]. Stem-cell-derived vessel-on-chip systems and tissue-engineered vascular aging models further suggest that cell-source selection and chronic oxidative or inflammatory conditioning can enhance disease relevance [43,44]. Broader reviews and commentaries emphasize that AS-on-chip models should combine vascular geometry, immune-cell migration, lipid handling, and hemodynamic stimulation rather than relying on cytokine exposure alone [45,46]. Pumpless or modular perfusion methods and dedicated vessel-on-chip inflammation protocols may also simplify long-term culture and improve reproducibility in future versions of this platform [47,48]. In parallel, cardiac or vascular organ-on-chip reviews and vascular barrier cell-source discussions support clearer reporting standards for cell origin, ECM composition, flow conditions, and endpoint assays [49,50].

## 5. Conclusions

We have developed a four-channel microfluidic vascular-wall chip that reconstitutes endothelial, extracellular matrix, and smooth muscle compartments, enabling low-flow modeling of inflammatory endothelial dysfunction. TNF-alpha/IL-1beta stimulation promoted macrophage recruitment, IL-6 and MCP-1 secretion, ZO-1 disruption, and increased FITC-dextran permeability. The combination of metformin and atorvastatin attenuated MCP-1 secretion more strongly than either monotherapy. The platform is best positioned as a microfluidic early vascular inflammation and endothelial barrier dysfunction model, with future development needed to incorporate arterial endothelial cells, lipid deposition, foam-cell formation, and controlled arterial shear.

## Figures and Tables

**Table 1:** Comparison of representative in vitro vascular inflammation and atherosclerosis-related models with the present four-channel vascular-wall chip.

Model/Study	Cellular/ECM Structure	Flow or Shear Condition	Immune or Lipid Component	Main Readouts	Relevance and Limitations
Static 2D culture	Single cell type or simple co-culture; no 3D ECM	No perfusion	Usually absent or endpoint-only	Cell viability, cytokines, adhesion assays	Simple and reproducible, but weak microenvironmental relevance
Transwell vascular inflammation models	Endothelial monolayer on porous membrane; optional SMC/macrophage compartment	Mostly static	Monocyte/macrophage migration can be assessed	TEER or tracer leakage	Useful for barrier readout, but limited spatial and flow control
AS-related organ-on-chip reviews [16]	3D microfluidic models with vessel-wall or plaque-related modules	Model-dependent	Immune and lipid modules vary	Permeability, imaging, cytokines	Clarifies design principles and current gaps
Human arterial wall-on-chip [17]	Endothelial and vascular smooth muscle compartments	Perfusable platform	Inflammation-triggered immune-related readouts	EC inflammation and SMC migration	Relevant early-AS model, but still simplified
3D AS-on-chip model [18]	Multicellular 3D arterial plaque-related architecture	Flow included	Immune cells and foam-cell-related modules included	3D imaging and arterial-event readouts	Stronger AS specificity but higher complexity
This four-channel vascular-wall chip	HUVECs, HASMC-laden collagen I, cell-free collagen I, and medium channel	Continuous low-flow perfusion; estimated low shear	THP-1-derived M0-like macrophage recruitment; no oxLDL or foam cells	ZO-1 imaging, FITC-dextran permeability, IL-6, MCP-1	Models early inflammatory endothelial dysfunction, not advanced plaque formation

## Data Availability

The data presented in this study are available in the article and from the corresponding author upon reasonable request.
